# Crystal structure of di-μ-iodido-bis­[(dimethyl sulfoxide-κ*O*)(tri­phenyl­phosphane-κ*P*)copper(I)]

**DOI:** 10.1107/S1600536814025203

**Published:** 2014-11-21

**Authors:** Rodolphe Kinghat, Michael Knorr, Yoann Rousselin, Marek M. Kubicki

**Affiliations:** a Institut UTINAM UMR CNRS 6213, University of Franche-Comté, 16 Route de Gray, Besançon, 25030, France; b ICMUB UMR CNRS 5260, University of Bourgogne, 9 Avenue A. Savary, Dijon, 21078, France

**Keywords:** crystal structure, dinuclear Cu^I^ complexes, iodide bridges, tri­phenyl­phosphane, DMSO

## Abstract

The centrosymmetric dinuclear title compound, [Cu_2_I_2_(C_2_H_6_OS)_2_(C_18_H_15_P)_2_], represents the first example of a CuI complex ligated by an *O*-bound dimethyl sulfoxide ligand. In the crystal, the two tetrahedrally coordinated Cu^I^ atoms are bridged by two μ_2_-iodido ligands in an almost symmetrical rhomboid geometry. The loose Cu⋯Cu contact of 2.9874 (8) Å is longer than the sum of the van der Waals radii of two Cu atoms (2.8 Å), excluding a significant cupriophilic inter­action in the actual dimer. C—H⋯O and C—H⋯I hydrogen bonding interactions as well as C—H⋯π(aryl) interactions stabilize the three-dimensional supramolecular network.

## Abstract 2

The basic building unit of the title complex, CuI(DMSO)(PPh_3_), reproduced by a symmetry centre, leads to the rhomboid dimers in which the Cu^I^ atoms are in a tetra­hedral geometry. The dimers are discrete mol­ecules, but through weak inter­molecular C—H⋯O inter­actions involving two adjacent DMSO ligands, a one-dimensional chain assembly is formed.

## Chemical context   

There exists a large family of dinuclear Cu^I^⋯Cu^I^-halide-bridged complexes of the type [PPh_3_(*L′*)Cu(μ_2_-I)_2_Cu(*L*′)PPh_3_], with the ligands *L* commonly bearing the coordinating N and S atoms, in which cupriophilic inter­actions may play a crucial role in determining their photophysical properties (Lobana *et al.*, 2012 ▶ and references therein; Engelhardt *et al.*, 1989 ▶). The title compound, [PPh_3_(DMSO)Cu(μ_2_-I)_2_Cu(DMSO)PPh_3_] (1), belongs to this family of compounds for which an association of *L* = PPh_3_ and *L*′ = DMSO has never been mentioned before.
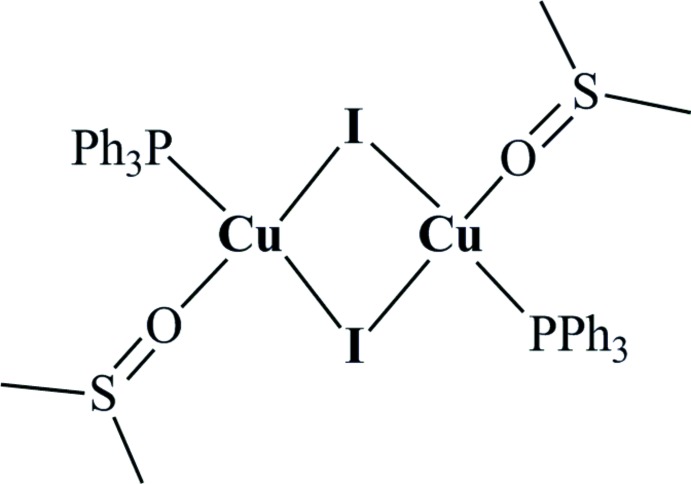


## Database survey   

The polar aprotic solvent (CH_3_)_2_S=O (DMSO) is frequently used in organic chemistry for reactions involving salts such nucleophilic substitutions reactions, but it has also found widespread use as a ligating solvent in the coordination chemistry of transition metals, where it may act both as an *S*-donor and an *O*-donor ligand towards a metal centre (Selbin *et al.*, 1961 ▶). A survey of the Cambridge Structural Database (CSD; Groom & Allen, 2014 ▶) reveals a large number of structurally characterized Cu^II^ halide complexes ligated by *O*-bound DMSO ligands. However, we found just one entry concerning a Cu^I^ halide complex, namely the tetra­metallic chain complex [Cu_4_Br(μ-Br)_3_(μ-dpmppm)_2_(DMSO)_2_] (dpmppm = bis­[(di­phenyl­phosphinometh­yl)phenyl­phos­phino]methane) reported by Takemura *et al.* (2009 ▶). Note that in the case of a soft Cu^I^ ion (compared with a harder Cu^II^ ion according the HSAB principle), DMSO could be *a priori* coordinating either *via* the sulfur or *via* the oxygen atom. Surprisingly, we found no Cu^I^I complex ligated by DMSO in the CSD.

## Structural commentary   

**Figure 1:**
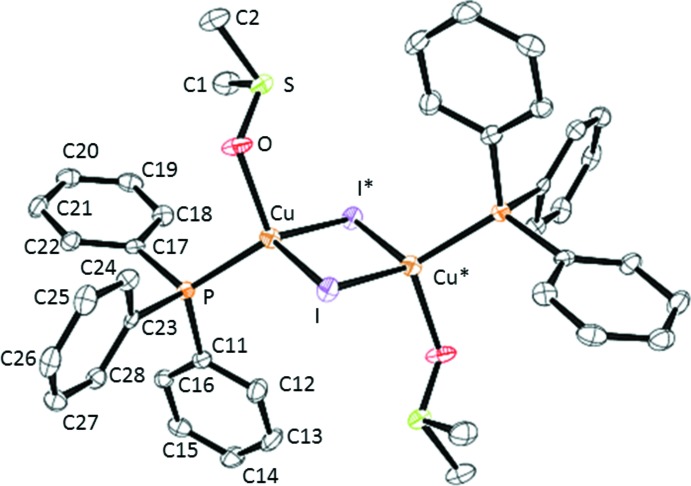
The mol­ecular structure of title compound built over a symmetry centre, with atom labels and 50% probability displacement ellipsoids for non-H atoms. Symmetry code for unlabelled atoms is (1 − *x*, −*y*, −*z*).

CuI is known to afford with DMSO in the presence of P_2_S_5_ the 2D coordination polymer [(Me_2_S)_3_{Cu_4_(μ-I)_4_}]_*n*_, the production of SMe_2_ being explained by the de­oxy­genation of Me_2_SO by P_2_S_5_ (Zhou *et al.*, 2006 ▶). In the context of our research on the coordination of thio­ethers *R*–S–*R* on Cu*X* salts (Knorr *et al.*, 2010 ▶; Lapprand *et al.*, 2013 ▶), we reacted a CuI solution in hot DMSO with a stoichiometric amount of PPh_3_ and succeeded in isolating in moderate yield X-ray-suitable crystals of (1). Structural analysis revealed that a centrosymmetric dinuclear complex is formed (Fig. 1 ▶), in which the two tetra­hedrally coordinated Cu^I^ atoms are bridged by two μ_2_-iodido ligands in a slightly asymmetric rhomboid manner. Despite the soft character of CuI, the DMSO ligands are *O*-bound. The Cu—O bond length of 2.140 (2) Å is considerably longer than those of polymeric Cu^II^ compounds [(DMSO)_2_CuBr_2_]_*n*_ [1.962 (9) Å; Willett *et al.*, 1977 ▶] and [(DMSO)_2_CuCl_2_]_*n*_ [1.955 (4) Å; Willett & Chang, 1970 ▶], but is in the same range as found for [Cu_4_Br(μ-Br)_3_(μ-dpmppm)_2_(DMSO)_2_] [2.200 (7) Å]. The Cu⋯Cu contact of 2.9874 (8) Å is longer than the sum of the van der Waals radii of two Cu atoms (2.8 Å), excluding any cupriophilic inter­action. This separation is in the same range as reported for [PPh_3_(pyridine)Cu(μ_2_-I)_2_Cu(pyridine)PPh_3_] (2.97 Å) (Bow­maker *et al.*, 1994 ▶), and the P—Cu bond lengths are also quite similar in the two compounds [2.2295 (10) *vs* 2.24 Å].

## Supra­molecular features   

**Figure 2:**
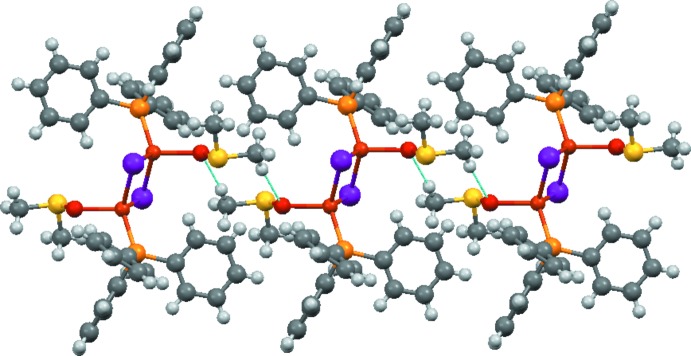
One-dimensional chain along [110] built *via* C—H⋯O inter­molecular inter­actions between the DMSO ligands.

The assembly of the crystal structure seems to be first governed by C—H⋯O-type hydrogen bonds (inter­molecular ligand-to-ligand DMSO inter­actions), leading to a 1D chain structure extending in the [110] direction (Fig. 2 ▶). Further, the very weak C—H⋯I inter­actions (for a 2D structure), followed by those of the C—H⋯π(ar­yl) type are probably responsible for the 3D assembly (Table 1 ▶).

## Synthesis and crystallization   

Tri­phenyl­phosphane (262 mg, 1.0 mmol) was added to a solution of CuI (192 mg, 1.0 mmol) in 10 ml of DMSO. The reaction mixture was first stirred at room temperature for 30 min and then heated for further 30 min to 368 K. After allowing the mixture to reach ambient temperature, yellowish crystals were formed (36% yield). Characterization data: ^1^H NMR (CDCl_3_): 2.62 (*s*, 6H, Me), 7.30–7.57 (*m*, 15H, Ph).

## Refinement   

Crystal data, data collection and structure refinement details are summarized in Table 2 ▶. All H atoms were placed in calculated positions and treated in a riding-model approximation. C—H distances were set to 0.95 (aromatic) and 0.98 Å (meth­yl) with *U_iso_*(H) = *xU_eq_*(C), where *x* = 1.5 for methyl and 1.2 for aromatic H atoms.

## Supplementary Material

Crystal structure: contains datablock(s) I, 08br35. DOI: 10.1107/S1600536814025203/gk2619sup1.cif

Structure factors: contains datablock(s) I. DOI: 10.1107/S1600536814025203/gk2619Isup2.hkl

CCDC reference: 1034622

Additional supporting information:  crystallographic information; 3D view; checkCIF report

## Figures and Tables

**Table 1:** Hydrogen-bond geometry (, )

*D*H*A*	*D*H	H*A*	*D* *A*	*D*H*A*
C2H2*A*O^i^	0.98	2.46	3.434(5)	173
C1H1*B*I^ii^	0.98	3.12	3.931(4)	142
C2H2*B*I^ii^	0.98	3.15	3.978(4)	143
C26H26C16^iii^	0.95	2.85	3.781(5)	168

Symmetry codes: (i) 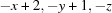; (ii) 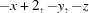; (iii) 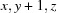.

**Table 2:** Experimental details

Crystal data	
Chemical formula	[Cu_2_I_2_(C_2_H_6_OS)_2_(C_18_H_15_P)_2_]
*M*_r_	1061.67
Crystal system, space group	Triclinic, *P* 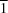
Temperature (K)	115
*a*, *b*, *c* ()	8.6099(2), 9.3435(2), 14.5279(4)
, , ()	91.016(1), 104.049(1), 116.004(1)
*V* (^3^)	1008.60(4)
*Z*	1
Radiation type	Mo *K*
(mm^1^)	2.80
Crystal size (mm)	0.17 0.05 0.05

Data collection	
Diffractometer	Nonius KappaCCD
No. of measured, independent and observed [*I* > 2(*I*)] reflections	8368, 4586, 3541
*R*_int_	0.036
(sin /)_max_ (^1^)	0.649

Refinement	
*R*[*F* ^2^ > 2(*F* ^2^)], *wR*(*F* ^2^), *S*	0.035, 0.077, 0.99
No. of reflections	4586
No. of parameters	228
H-atom treatment	H-atom parameters constrained
_max_, _min_ (e ^3^)	0.78, 0.98

Computer programs: *DENZO* and *SCALEPACK* (Otwinowski Minor, 1997 ▶), *SIR97* (Altomare *et al.*, 1999 ▶), *SHELXL2012* (Sheldrick, 2008 ▶), *ORTEP-3 for Windows* and *WinGX* (Farrugia, 2012 ▶).

## supplementary crystallographic information

### Crystal data

**Table:**

[Cu_2_I_2_(C_2_H_6_OS)_2_(C_18_H_15_P)_2_]	*Z* = 1
*M**_r_* = 1061.67	*F*(000) = 524
Triclinic, *P*1	*D*_x_ = 1.748 Mg m^−^^3^
*a* = 8.6099 (2) Å	Mo *K*α radiation, λ = 0.71073 Å
*b* = 9.3435 (2) Å	Cell parameters from 8449 reflections
*c* = 14.5279 (4) Å	θ = 1.0–27.5°
α = 91.016 (1)°	µ = 2.80 mm^−^^1^
β = 104.049 (1)°	*T* = 115 K
γ = 116.004 (1)°	Prism, clear light colourless
*V* = 1008.60 (4) Å^3^	0.17 × 0.05 × 0.05 mm

### Data collection

**Table:**

Nonius KappaCCD diffractometer	3541 reflections with *I* > 2σ(*I*)
Radiation source: X-ray tube, Enraf–Nonius FR590	*R*_int_ = 0.036
Horizonally mounted graphite crystal monochromator	θ_max_ = 27.5°, θ_min_ = 2.9°
Detector resolution: 9 pixels mm^-1^	*h* = −11→11
CCD rotation images, thick slices scans	*k* = −12→12
8368 measured reflections	*l* = −18→18
4586 independent reflections	

### Refinement

**Table:**

Refinement on *F*^2^	Primary atom site location: structure-invariant direct methods
Least-squares matrix: full	Secondary atom site location: difference Fourier map
*R*[*F*^2^ > 2σ(*F*^2^)] = 0.035	Hydrogen site location: inferred from neighbouring sites
*wR*(*F*^2^) = 0.077	H-atom parameters constrained
*S* = 0.99	*w* = 1/[σ^2^(*F*_o_^2^) + (0.0332*P*)^2^] where *P* = (*F*_o_^2^ + 2*F*_c_^2^)/3
4586 reflections	(Δ/σ)_max_ = 0.001
228 parameters	Δρ_max_ = 0.78 e Å^−^^3^
0 restraints	Δρ_min_ = −0.98 e Å^−^^3^

### Special details

**Table:**

Geometry. All e.s.d.'s (except the e.s.d. in the dihedral angle between two l.s. planes) are estimated using the full covariance matrix. The cell e.s.d.'s are taken into account individually in the estimation of e.s.d.'s in distances, angles and torsion angles; correlations between e.s.d.'s in cell parameters are only used when they are defined by crystal symmetry. An approximate (isotropic) treatment of cell e.s.d.'s is used for estimating e.s.d.'s involving l.s. planes.

### Fractional atomic coordinates and isotropic or equivalent isotropic displacement parameters (Å^2^)

**Table:**

	*x*	*y*	*z*	*U*_iso_*/*U*_eq_	
C1	1.1097 (5)	0.1762 (5)	0.1426 (3)	0.0287 (9)	
H1A	1.2180	0.1767	0.1319	0.043*	
H1B	1.0227	0.0656	0.1443	0.043*	
H1C	1.1417	0.2424	0.2038	0.043*	
C2	1.2026 (5)	0.4468 (4)	0.0613 (3)	0.0289 (10)	
H2A	1.2436	0.4995	0.1276	0.043*	
H2B	1.1687	0.5138	0.0179	0.043*	
H2C	1.2999	0.4324	0.0458	0.043*	
C11	0.4456 (4)	−0.0357 (4)	0.2784 (3)	0.0164 (8)	
C12	0.2884 (5)	−0.1295 (4)	0.2069 (3)	0.0261 (9)	
H12	0.2763	−0.1044	0.1432	0.031*	
C13	0.1487 (5)	−0.2598 (4)	0.2276 (3)	0.0309 (10)	
H13	0.0407	−0.3222	0.1784	0.037*	
C14	0.1662 (5)	−0.2989 (4)	0.3195 (3)	0.0270 (9)	
H14	0.0711	−0.3890	0.3335	0.032*	
C15	0.3233 (5)	−0.2061 (4)	0.3913 (3)	0.0228 (8)	
H15	0.3355	−0.2321	0.4548	0.027*	
C16	0.4625 (5)	−0.0756 (4)	0.3707 (3)	0.0178 (8)	
H16	0.5703	−0.0130	0.4199	0.021*	
C17	0.8273 (4)	0.1580 (4)	0.3337 (3)	0.0154 (7)	
C18	0.8942 (5)	0.0488 (4)	0.3218 (3)	0.0193 (8)	
H18	0.8316	−0.0358	0.2693	0.023*	
C19	1.0498 (5)	0.0623 (4)	0.3852 (3)	0.0235 (9)	
H19	1.0937	−0.0127	0.3763	0.028*	
C20	1.1422 (5)	0.1862 (4)	0.4623 (3)	0.0244 (9)	
H20	1.2490	0.1959	0.5063	0.029*	
C21	1.0783 (5)	0.2938 (4)	0.4742 (3)	0.0240 (9)	
H21	1.1414	0.3784	0.5267	0.029*	
C22	0.9227 (4)	0.2810 (4)	0.4107 (3)	0.0187 (8)	
H22	0.8807	0.3573	0.4198	0.022*	
C23	0.5978 (4)	0.3083 (4)	0.2747 (2)	0.0145 (7)	
C24	0.6736 (5)	0.4388 (4)	0.2269 (3)	0.0201 (8)	
H24	0.7384	0.4338	0.1835	0.024*	
C25	0.6536 (5)	0.5769 (4)	0.2431 (3)	0.0236 (9)	
H25	0.7075	0.6670	0.2119	0.028*	
C26	0.5561 (5)	0.5829 (4)	0.3040 (3)	0.0229 (9)	
H26	0.5403	0.6760	0.3131	0.028*	
C27	0.4813 (5)	0.4551 (4)	0.3520 (3)	0.0219 (8)	
H27	0.4160	0.4612	0.3948	0.026*	
C28	0.5013 (4)	0.3171 (4)	0.3378 (3)	0.0180 (8)	
H28	0.4496	0.2290	0.3708	0.022*	
O	0.8790 (3)	0.2915 (3)	0.0820 (2)	0.0270 (6)	
P	0.62437 (12)	0.13151 (10)	0.24350 (7)	0.0145 (2)	
S	1.01292 (12)	0.25565 (10)	0.04801 (7)	0.0206 (2)	
Cu	0.62834 (6)	0.10410 (5)	0.09145 (3)	0.01942 (12)	
I	0.60513 (3)	−0.17153 (2)	0.03064 (2)	0.02049 (9)	

### Atomic displacement parameters (Å^2^)

**Table:**

	*U*^11^	*U*^22^	*U*^33^	*U*^12^	*U*^13^	*U*^23^
C1	0.023 (2)	0.038 (2)	0.028 (2)	0.0175 (19)	0.0055 (18)	0.0123 (18)
C2	0.0155 (19)	0.023 (2)	0.045 (3)	0.0060 (16)	0.0084 (18)	0.0055 (18)
C11	0.0143 (18)	0.0132 (17)	0.022 (2)	0.0066 (14)	0.0051 (15)	0.0019 (14)
C12	0.024 (2)	0.027 (2)	0.018 (2)	0.0044 (17)	0.0029 (17)	0.0028 (16)
C13	0.016 (2)	0.029 (2)	0.031 (3)	−0.0013 (17)	0.0020 (18)	−0.0011 (18)
C14	0.022 (2)	0.0182 (19)	0.035 (3)	0.0016 (16)	0.0126 (18)	0.0056 (17)
C15	0.026 (2)	0.024 (2)	0.025 (2)	0.0136 (17)	0.0135 (18)	0.0091 (16)
C16	0.0168 (18)	0.0154 (18)	0.021 (2)	0.0070 (15)	0.0063 (15)	0.0023 (15)
C17	0.0111 (17)	0.0169 (18)	0.018 (2)	0.0052 (14)	0.0061 (15)	0.0048 (14)
C18	0.0224 (19)	0.0148 (17)	0.022 (2)	0.0087 (15)	0.0071 (16)	0.0064 (15)
C19	0.024 (2)	0.027 (2)	0.030 (2)	0.0174 (17)	0.0132 (18)	0.0116 (17)
C20	0.0179 (19)	0.032 (2)	0.024 (2)	0.0124 (17)	0.0052 (17)	0.0087 (17)
C21	0.0163 (19)	0.0224 (19)	0.025 (2)	0.0049 (16)	−0.0009 (16)	−0.0038 (16)
C22	0.0152 (18)	0.0184 (18)	0.022 (2)	0.0087 (15)	0.0026 (16)	0.0017 (15)
C23	0.0095 (16)	0.0136 (17)	0.0149 (19)	0.0030 (14)	−0.0016 (14)	−0.0017 (14)
C24	0.0180 (19)	0.0182 (18)	0.023 (2)	0.0073 (15)	0.0049 (16)	0.0033 (15)
C25	0.029 (2)	0.0147 (18)	0.022 (2)	0.0084 (16)	0.0022 (17)	0.0042 (15)
C26	0.024 (2)	0.0166 (19)	0.026 (2)	0.0151 (16)	−0.0068 (17)	−0.0038 (16)
C27	0.0200 (19)	0.024 (2)	0.025 (2)	0.0128 (16)	0.0060 (17)	−0.0005 (16)
C28	0.0162 (18)	0.0177 (18)	0.019 (2)	0.0076 (15)	0.0031 (15)	0.0024 (15)
O	0.0147 (13)	0.0222 (13)	0.0462 (19)	0.0076 (11)	0.0138 (12)	0.0082 (12)
P	0.0139 (4)	0.0123 (4)	0.0175 (5)	0.0062 (4)	0.0042 (4)	0.0020 (4)
S	0.0147 (5)	0.0205 (5)	0.0232 (5)	0.0059 (4)	0.0036 (4)	0.0038 (4)
Cu	0.0195 (2)	0.0204 (2)	0.0200 (3)	0.0100 (2)	0.0066 (2)	0.00351 (19)
I	0.02019 (14)	0.01809 (13)	0.02255 (15)	0.01034 (10)	0.00187 (10)	0.00121 (10)

### Geometric parameters (Å, º)

**Table:**

C1—H1A	0.9800	C19—C20	1.393 (5)
C1—H1B	0.9800	C20—H20	0.9500
C1—H1C	0.9800	C20—C21	1.367 (5)
C1—S	1.777 (4)	C21—H21	0.9500
C2—H2A	0.9800	C21—C22	1.384 (5)
C2—H2B	0.9800	C22—H22	0.9500
C2—H2C	0.9800	C23—C24	1.396 (5)
C2—S	1.781 (3)	C23—C28	1.401 (5)
C11—C12	1.387 (5)	C23—P	1.828 (3)
C11—C16	1.388 (5)	C24—H24	0.9500
C11—P	1.834 (3)	C24—C25	1.398 (4)
C12—H12	0.9500	C25—H25	0.9500
C12—C13	1.386 (5)	C25—C26	1.374 (5)
C13—H13	0.9500	C26—H26	0.9500
C13—C14	1.382 (5)	C26—C27	1.379 (5)
C14—H14	0.9500	C27—H27	0.9500
C14—C15	1.387 (5)	C27—C28	1.392 (4)
C15—H15	0.9500	C28—H28	0.9500
C15—C16	1.386 (5)	O—S	1.514 (2)
C16—H16	0.9500	O—Cu	2.140 (2)
C17—C18	1.399 (4)	P—Cu	2.2295 (10)
C17—C22	1.388 (5)	Cu—Cu^i^	2.9874 (8)
C17—P	1.825 (3)	Cu—I	2.6144 (4)
C18—H18	0.9500	Cu—I^i^	2.6463 (5)
C18—C19	1.381 (5)	I—Cu^i^	2.6463 (5)
C19—H19	0.9500		
			
H1A—C1—H1B	109.5	C22—C21—H21	119.6
H1A—C1—H1C	109.5	C17—C22—H22	119.6
H1B—C1—H1C	109.5	C21—C22—C17	120.8 (3)
S—C1—H1A	109.5	C21—C22—H22	119.6
S—C1—H1B	109.5	C24—C23—C28	119.5 (3)
S—C1—H1C	109.5	C24—C23—P	115.5 (3)
H2A—C2—H2B	109.5	C28—C23—P	124.9 (3)
H2A—C2—H2C	109.5	C23—C24—H24	120.2
H2B—C2—H2C	109.5	C23—C24—C25	119.6 (3)
S—C2—H2A	109.5	C25—C24—H24	120.2
S—C2—H2B	109.5	C24—C25—H25	119.9
S—C2—H2C	109.5	C26—C25—C24	120.3 (3)
C12—C11—C16	119.1 (3)	C26—C25—H25	119.9
C12—C11—P	117.3 (3)	C25—C26—H26	119.7
C16—C11—P	123.6 (3)	C25—C26—C27	120.6 (3)
C11—C12—H12	119.8	C27—C26—H26	119.7
C13—C12—C11	120.5 (4)	C26—C27—H27	120.0
C13—C12—H12	119.8	C26—C27—C28	120.0 (3)
C12—C13—H13	119.9	C28—C27—H27	120.0
C14—C13—C12	120.2 (4)	C23—C28—H28	120.0
C14—C13—H13	119.9	C27—C28—C23	119.9 (3)
C13—C14—H14	120.1	C27—C28—H28	120.0
C13—C14—C15	119.7 (4)	S—O—Cu	121.91 (13)
C15—C14—H14	120.1	C11—P—Cu	116.27 (11)
C14—C15—H15	120.0	C17—P—C11	102.77 (16)
C16—C15—C14	120.1 (4)	C17—P—C23	103.83 (14)
C16—C15—H15	120.0	C17—P—Cu	115.71 (12)
C11—C16—H16	119.8	C23—P—C11	104.58 (15)
C15—C16—C11	120.4 (3)	C23—P—Cu	112.25 (12)
C15—C16—H16	119.8	C1—S—C2	98.10 (19)
C18—C17—P	117.0 (3)	O—S—C1	106.07 (17)
C22—C17—C18	118.1 (3)	O—S—C2	104.70 (16)
C22—C17—P	124.9 (3)	O—Cu—P	106.70 (8)
C17—C18—H18	119.5	O—Cu—Cu^i^	116.74 (8)
C19—C18—C17	121.0 (3)	O—Cu—I	108.23 (6)
C19—C18—H18	119.5	O—Cu—I^i^	101.47 (7)
C18—C19—H19	120.1	P—Cu—Cu^i^	136.38 (3)
C18—C19—C20	119.8 (3)	P—Cu—I^i^	114.07 (3)
C20—C19—H19	120.1	P—Cu—I	114.48 (3)
C19—C20—H20	120.2	I—Cu—Cu^i^	55.904 (14)
C21—C20—C19	119.6 (3)	I^i^—Cu—Cu^i^	54.897 (14)
C21—C20—H20	120.2	I—Cu—I^i^	110.801 (16)
C20—C21—H21	119.6	Cu—I—Cu^i^	69.201 (16)
C20—C21—C22	120.8 (3)		
			
C11—C12—C13—C14	−1.1 (6)	C22—C17—P—C23	4.7 (4)
C12—C11—C16—C15	−0.9 (5)	C22—C17—P—Cu	128.1 (3)
C12—C11—P—C17	−154.0 (3)	C23—C24—C25—C26	−1.6 (5)
C12—C11—P—C23	97.8 (3)	C24—C23—C28—C27	0.2 (5)
C12—C11—P—Cu	−26.6 (3)	C24—C23—P—C11	−158.7 (3)
C12—C13—C14—C15	0.8 (6)	C24—C23—P—C17	93.8 (3)
C13—C14—C15—C16	−0.5 (5)	C24—C23—P—Cu	−31.8 (3)
C14—C15—C16—C11	0.6 (5)	C24—C25—C26—C27	1.9 (5)
C16—C11—C12—C13	1.2 (5)	C25—C26—C27—C28	−1.1 (5)
C16—C11—P—C17	23.6 (3)	C26—C27—C28—C23	0.1 (5)
C16—C11—P—C23	−84.6 (3)	C28—C23—C24—C25	0.5 (5)
C16—C11—P—Cu	151.0 (2)	C28—C23—P—C11	17.9 (3)
C17—C18—C19—C20	0.0 (5)	C28—C23—P—C17	−89.5 (3)
C18—C17—C22—C21	−0.7 (5)	C28—C23—P—Cu	144.8 (3)
C18—C17—P—C11	78.3 (3)	P—C11—C12—C13	178.8 (3)
C18—C17—P—C23	−172.9 (3)	P—C11—C16—C15	−178.4 (2)
C18—C17—P—Cu	−49.5 (3)	P—C17—C18—C19	178.3 (3)
C18—C19—C20—C21	−0.3 (6)	P—C17—C22—C21	−178.3 (3)
C19—C20—C21—C22	0.1 (6)	P—C23—C24—C25	177.4 (3)
C20—C21—C22—C17	0.4 (6)	P—C23—C28—C27	−176.3 (2)
C22—C17—C18—C19	0.5 (5)	Cu—O—S—C1	−72.7 (2)
C22—C17—P—C11	−104.1 (3)	Cu—O—S—C2	−175.87 (19)

Symmetry code: (i) −*x*+1, −*y*, −*z*.

### Hydrogen-bond geometry (Å, º)

**Table:**

*D*—H···*A*	*D*—H	H···*A*	*D*···*A*	*D*—H···*A*
C2—H2*A*···O^ii^	0.98	2.46	3.434 (5)	173
C1—H1*B*···I^iii^	0.98	3.12	3.931 (4)	142
C2—H2*B*···I^iii^	0.98	3.15	3.978 (4)	143
C26—H26···C16^iv^	0.95	2.85	3.781 (5)	168

Symmetry codes: (ii) −*x*+2, −*y*+1, −*z*; (iii) −*x*+2, −*y*, −*z*; (iv) *x*, *y*+1, *z*.
